# No correlation between thrombin generation and emicizumab levels: implications for monitoring emicizumab therapy

**DOI:** 10.1016/j.rpth.2024.102658

**Published:** 2024-12-17

**Authors:** Konrad van der Zwet, Mark Roest, Dana Huskens, Roger E.G. Schutgens, Lize F.D. van Vulpen, Kathelijn Fischer, Rolf T. Urbanus

**Affiliations:** 1 Center for Benign Haematology, Thrombosis and Haemostasis, Van Creveldkliniek, University Medical Center Utrecht, Utrecht University, Utrecht, The Netherlands; 2 Synapse Research Institute, Maastricht, The Netherlands; 3 Pednet Haemophilia Research Foundation, Baarn, The Netherlands

**Keywords:** drug monitoring, emicizumab, hemophilia A, monoclonal antibodies, thrombin generation

## Abstract

**Background:**

Emicizumab, a bispecific antibody that mimics factor (F)VIII, has significantly improved hemophilia A management. Although emicizumab levels can be measured, tools for estimating the hemostatic efficacy of emicizumab are lacking. Thrombin generation (TG) assays can distinguish bleeding phenotypes in persons with hemophilia A on FVIII prophylaxis and may also be used during emicizumab therapy.

**Objectives:**

To assess the association between TG parameters, emicizumab levels, and bleeding in patients on emicizumab therapy.

**Methods:**

A single-center longitudinal cohort study was conducted, with samples collected during the steady-state phase of emicizumab therapy. TG was measured using tissue factor (TF; TF-TG, 1 pM) and FXIa (FXIa-TG, 200 pM). Emicizumab concentrations were determined with mass spectrometry. Only treated bleeds were recorded. Pearson correlations (rho, *r*) were reported.

**Results:**

Eighty-five samples from 49 patients were analyzed during a median of 1 year of emicizumab therapy. Most bleeds were traumatic (97%; *n* = 30), whereas 1 bleed was spontaneous. At 12 months, TF-TG (*r* = 0.42) showed a borderline correlation, and FXIa-TG (*r* = 0.15) showed no correlation with emicizumab concentrations. Although FXIa-TG showed a 9% higher endogenous thrombin potential in patients with zero vs ≥1 treated bleed (endogenous thrombin potential: 957 vs 878 nM/min, *P* = .045), neither the FXIa-peak height nor TF-TG showed any association with traumatic bleeding.

**Conclusion:**

TG parameters showed no clinically relevant correlations with emicizumab plasma concentrations, were not associated with traumatic bleeding, and showed considerable intrapatient variability. Therefore, TG was not considered useful for monitoring coagulation potential in patients on steady-state emicizumab prophylaxis.

## Essentials

• Emicizumab improved hemophilia A care but interferes with most standard coagulation tests.
• A longitudinal study evaluated TG parameters in patients on emicizumab.
• TG parameters showed no clinically relevant correlation with emicizumab plasma levels.
• TG parameters showed considerable intrapatient variability.

## 1 Introduction

Emicizumab (Hemlibra, Roche) is a bispecific antibody that binds activated factor (F)IXa and FX, allowing activation of FX and subsequent thrombin generation (TG) [1]. Emicizumab was demonstrated to reduce the annualized (joint) bleeding rate (A[J]BR) in persons with hemophilia A (HA), both with and without inhibitors, in comparison with previous therapeutic regimens [2]. Emicizumab is administered at a fixed dose based on body weight, and monitoring is not required according to the drug label [3].

Although emicizumab is effective in reducing the A(J)BR, spontaneous breakthrough bleeds and thrombosis have been reported in patients on emicizumab therapy [4]. Therefore, clinicians would benefit from a diagnostic test that is able to monitor hemostatic activity in patients on steady-state emicizumab therapy. Currently, there are no routine laboratory tests available to monitor the efficacy of emicizumab. Since emicizumab interferes with standard coagulation tests that measure the intrinsic pathway, including activated partial thromboplastin time and one-step FVIII activity assays, these tests are unsuitable for monitoring its effects.

The TG assay measures thrombin over time rather than clot formation and provides more information on activation and inhibition of coagulation than traditional clotting tests. Importantly, TG assays have enabled the differentiation of various bleeding phenotypes in persons with HA treated with FVIII replacement therapy based on endogenous thrombin potential (ETP) [[5], [6], [7]]. The ETP can be accurately measured in the presence of emicizumab; therefore, it may be a useful tool for monitoring emicizumab therapy.

Several studies have explored the relationship between TG parameters and emicizumab concentration or bleeding risk, but data are inconclusive [[8], [9], [10]]. Data from the HAVEN 1 study showed a linear relationship between emicizumab concentration and thrombin peak [8]. In contrast, results from a smaller study (*n* = 56) reported the absence of a dose-effect relationship between emicizumab concentration and TG [9]. Similar disparities were reported regarding the relationship between TG parameters and bleeding, with one study (*n* = 107) reporting a lack of correlation between tissue factor (TF)-initiated TG and bleeding within 12 months of initiating emicizumab and another study reporting lower ETP in patients with spontaneous bleeding [9,10].

The aim of this longitudinal cohort study was to investigate the association between ETP and emicizumab plasma concentration in persons with HA on steady-state emicizumab prophylaxis.

## 2 Methods

### 2.1 Study design and population

Samples were obtained from an ongoing prospective single-center cohort study collecting blood samples from patients switching to emicizumab therapy. All participants were recruited during routine visits and provided informed consent. Blood samples were collected at fixed time points during loading and at 3 (T3) and 12 months (T12) of emicizumab therapy.

The study was approved by the local Medical Research Ethics Committee. All patients were administered emicizumab in accordance with the product label (ie, after loading a maintenance dose of 6 mg/kg/4 wk, with varying intervals to avoid discarding emicizumab) [11,12].

The primary objective of the present study was to evaluate the association between TG parameters and emicizumab plasma levels in persons with HA at steady-state emicizumab prophylaxis. Secondary objectives were related to the TG parameters and bleeding control of emicizumab therapy.

### 2.2 Sampling and measurement

Samples were obtained during the steady state of emicizumab therapy at T3 and T12 after initiation. Blood was obtained by venepuncture in tubes containing sodium citrate (3.8%). Platelet-poor plasma was prepared by centrifugation at 2000 × *g* for 10 minutes and stored in aliquots of 250 μL at −80 °C until use.

TG was measured with calibrated automated thrombinography (Stago) as described, with a few modifications [13]. In short, TG was initiated with TF (1 pM, PPP Low Reagent [Stago]) according to the instructions of the manufacturer or with FXIa (200 pM, Synapse Research Institute). Hereto, FXIa was mixed with phospholipids (4 μM final concentration) and incubated with plasma for 10 minutes prior to initiation of TG with FluCa Reagent (Stago). Data were analyzed with Thrombinoscope Calibrated Automated Thrombinography software. TG parameters ETP and thrombin peak height were reported. The interassay variability (*n* = 6) for the TF-initiated TG in normal pooled plasma was 5.82% and 7.50% for ETP and peak height, respectively, and for the FXIa-initiated TG, it was 3.55% and 3.59% for ETP and peak height, respectively. Emicizumab concentrations were determined in parallel using a validated liquid-chromatography tandem mass spectrometry method as described [14].

### 2.3 Data collection

Clinical data were extracted from electronic patient records. Bleeding data were collected from electronic patient records and/or electronic patient diaries. Only bleeds treated with FVIII concentrates or bypassing agents were recorded, including both traumatic and spontaneous bleeds.

### 2.4 Statistical analysis

Patient characteristics were summarized as numbers (%) and medians with IQR (P25-P75). The relationship between emicizumab plasma concentrations and TG parameters was analyzed using Pearson’s correlation (rho, *r*). Correlation coefficients were classified as very strong (≥0.8), strong (0.60-0.79), and moderate (0.40-0.59), and lower correlations were considered clinically irrelevant according to established guidelines [15]. Changes over time for TG parameters and emicizumab concentrations were analyzed using multilevel regression analysis. To account for variations in follow-up duration and the skewed distribution of the data, the mean annualized bleeding rate and A(J)BR with their 95% CIs were modeled using negative binomial regression [16]. The Wilcoxon sum rank test was used to compare TG parameters between patients with and without bleeds. Statistical analyses were performed with R Studio (version R4.3.1, R Development Core Team).

## 3 Results and Discussion

A total of 49 persons with HA were included in the study (Table 1). The median age was 26 (range, 0-74 years) years, including 16 children (33% of the cohort). Most patients had severe HA (92%; *n* = 45). Two patients had active FVIII inhibitors (4%). The median emicizumab plasma concentration was 70.3 μg/mL (IQR, 58.0-79.9 μg/mL) at T3 and 66.8 μg/mL (IQR, 56.7-77.6 μg/mL) at T12 of therapy. Six patients (13%) had relevant comorbidities, including 3 patients with a history of cardiovascular disease and 3 patients with advanced liver cirrhosis.

**Table 1:** Baseline characteristics.

Baseline characteristics	Overall (*N* = 49)
Age at start of emicizumab therapy (y)	26 (15-48)
Follow-up on emicizumab therapy (y)	1.08 (1.03-1.14)
Hemophilia severity	
Severe	45 (92)
Moderate	4 (8)
Inhibitor history	
No FVIII inhibitor	47 (96)
FVIII inhibitor	2 (4)
History of cardiovascular disease	
No history of CVD	46 (94)
History of CVD	3 (6)
Advanced liver cirrhosis	
No advanced liver cirrhosis	46 (94)
Advanced liver cirrhosis	3 (6)

Results are presented as number (%) or median (P25-P75).

CVD, cardiovascular disease; FVIII, factor VIII.

**Figure 1:**
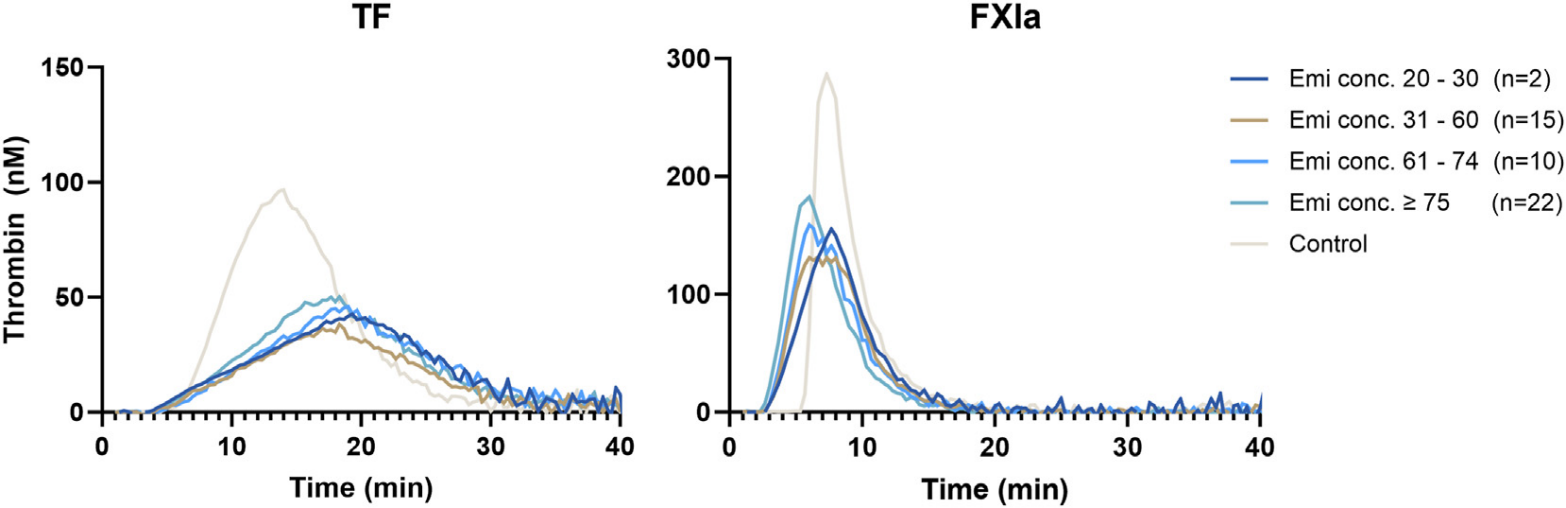
Mean thrombin generation curve for both thrombin generation initiated with tissue factor (TF; 1 pM) and factor (F)XIa (200 pM) according to emicizumab (Emi) plasma concentration.

A total of 44 samples were analyzed with TG at T3, and 41 samples were analyzed at T12 of emicizumab therapy; longitudinal samples were available for 36 patients (73%; Table 2). TG kinetics differed substantially between TF- and FXIa-initiated TG, with much sharper and higher peaks for FXIa compared with TF (Figure 1). Despite the intrapatient variability, TF-initiated TG parameters were stable over time (mean difference [IQR] for ETP, 63 nM ∗ min; *P* = .285; for peak height, 3 nM; *P* = .62), whereas ETP and peak height initiated with FXIa declined over time (mean difference [IQR] for ETP, 106 nM ∗ min; *P* < .001; for peak height, 12 nM; *P* = .07). Furthermore, we observed a trend toward lower TG parameters in children (<18 years) than adults, see Supplementary Table 1. However, we only observed a significant decrease for ETP at T3 initiated with FXIa, and for ETP and peak at T12 initiated with TF, as detailed in Supplementary Table 1.

**Table 2:** Median and IQRs for all thrombin generation parameters triggered with tissue factor and factor XIa at 3 months and 12 months of emicizumab therapy.

Thrombin generation parameters at 3 and 12 months of emicizumab therapy	TF-initiated TG		FXia-initiated TG	
	3 mo (*n* = 44)	12 mo (*n* = 41)	3 mo (*n* = 44)	12 mo (*n* = 41)
ETP (nM × min)	896 (622-1012)	757 (637-883)	1010 (923-1100)	887 (831-990)
Peak height (nM)	49 (32-73)	51 (30-67)	195 (167-227)	186 (159-213)
Time to peak (min)	19 (18-21)	19 (17-21)	7 (6-8)	7 (6-8)
Lag time (min)	6.3 (5.7-7.4)	6.5 (5.7-7.0)	3.4 (3.0-3.8)	3.3 (2.9-3.7)
Velocity index (nM/min)	3.7 (2.2-6.2)	4.1 (2.3-6.5)	60.6 (41.5-79.1)	57.3 (39.1-71.0)

ETP, endogenous thrombin potential; FXIa, factor XIa; TF, tissue factor; TG, thrombin generation.

Overall, we observed slight changes in TF-initiated TG with increasing emicizumab concentration, but no changes were observed in FXIa-initiated TG (Figure 1). Furthermore, statistical analysis showed that neither TG initiated with TF nor FXIa correlated with emicizumab concentration after T3 of therapy (Supplementary Figures 1 and 2). After T12 of therapy, the correlation between TF-initiated TG parameters and emicizumab concentration was borderline positive (ETP: *r* = 0.42 and peak: *r* = 0.41). For FXIa-initiated TG, ETP showed no correlation, and peak height only showed a borderline positive correlation (*r* = 0.40) with emicizumab concentration. Furthermore, no clinically relevant correlations were observed between TG parameters and emicizumab concentration in the subgroup analysis for children and adults.

Our findings are consistent with the French study that found no correlation between ETP and emicizumab concentration [9]. While the HAVEN 1 trial reported a linear association of FXIa-peak height with emicizumab concentrations, this was not corroborated by our findings. Unfortunately, the HAVEN 1 study only reported FXIa-initiated TG [8]. From a clinical point of view, TG measured with TF seems more relevant as it provides a more physiological assessment by incorporating all enzymatic reactions of the coagulation system, whereas FXIa specifically activates the intrinsic pathway. In contrast with previous studies suggesting it was a useful tool for dose adjustments in cases of breakthrough bleeding on emicizumab, we identified several limitations of TG [17,18].

First, despite a robust coefficient of variation for both TF- and FXIa-initiated TG, standardized sample processing, and TG assessment on the same day, significant intrapatient variability was observed in our longitudinal samples. This intrapatient variability cannot be explained by fluctuation between trough and peak emicizumab levels, as emicizumab concentrations were similar between these time points (mean difference between T3 and T12, 1.3 μg/mL; *P* = .587). Nor can variation be explained by changes in dosing regimens, as these have remained unchanged. Furthermore, changes in ETP and peak height with increasing emicizumab concentrations are minimal, suggesting that coagulation activation may already be maximized at the high emicizumab levels achieved with standard dosing regimens. Furthermore, these small differences in TG parameters we observed are likely not clinically relevant. However, it may still be valuable to explore TG in patients with emicizumab plasma concentrations in the lower region.

**Figure 2:**
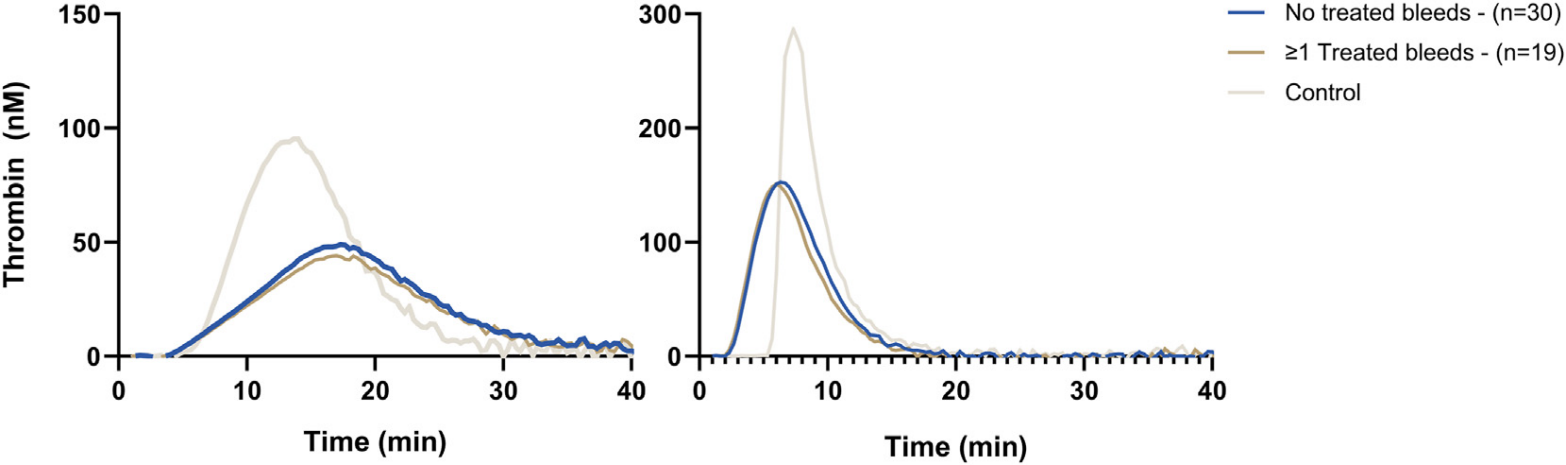
Mean thrombin generation curve for both thrombin generation initiated with tissue factor (1 pM) and factor XIa (200 pM) according to bleeding status.

Over a median duration of 1.08 years (IQR, 1.03-1.14 years) on emicizumab prophylaxis, we observed similar TG potential between patients without and with treated bleeds (Figure 2) with TF-initiated TG (ETP: 790 vs 719 nM/min, *P* = .418 and peak height: 54 vs 46 nM, *P* = .348). Although the ETP with FXIa-initiated TG showed a marginally higher ETP (+9%) in patients without treated bleeds (ETP: 957 vs 878 nM/min, *P* = .045), the difference was small, and the peak height was equal (183 vs 18 6nM, *P* = .84). Furthermore, the analysis of the association between bleeding and TG was hampered by the absence of bleeding (61% no treated bleeds) and, specifically, the absence of spontaneous bleeds (*n* = 1 only), which are more likely to correspond with coagulation potential than traumatic bleeds (Supplementary Table 2). As expected, emicizumab concentrations were similar between patients without and with treated bleeds at 69.4 vs 65.9 μg/mL, respectively (*P* = .539).

Our findings align with those from the Israeli study (*n* = 107), which also showed that peak height was not associated with bleeding. Moreover, they observed significant variability in TG, similar to our findings [10]. In contrast to our findings, the French study (*n* = 54) showed significantly lower ETP (−31.5%) in patients with a history of spontaneous bleeding during emicizumab therapy (1389 vs 951 nM/min, *P* < .001) [9].

While TF-initiated TG parameters showed greater stability over time than FXIa-initiated TG, we identified 3 patients who had a TF-ETP below 142 nM/min (5th percentile) at T3 of emicizumab therapy, with a median emicizumab concentration of 70.6 μg/mL (range, 57.7-77.5 μg/mL). Among these patients, 2 had longitudinal samples available, both of which showed normalized ETP values (645 and 740 nM/min, respectively) after 1 year of emicizumab therapy without any dose adjustments, further emphasizing the significant intrapatient variability in TG parameters.

The strength of our study lies in the longitudinal sampling of 36 patients, enabling measurement of TG both at T3 and T12 of steady-state emicizumab therapy. Additionally, both TF and FXIa triggers were utilized, enabling a direct comparison. Additionally, A(J)BRs were computed using negative binomial regression techniques that adjust for differences in the follow-up period of emicizumab therapy [16]. Limitations arise from the small sample size, with a limited number of bleeding events and only 1 spontaneous bleed. Furthermore, the study is limited by the absence of patients who developed antidrug antibodies against emicizumab, which may affect the generalizability of the findings for such cases.

In conclusion, this study demonstrated no clinically relevant correlations between TG parameters and emicizumab concentrations after T12 of emicizumab therapy. TG parameters were not associated with traumatic bleeding and exhibited significant intrapatient variability. Therefore, TG appears to be unsuitable for monitoring coagulation potential in patients on steady-state emicizumab prophylaxis.

## Funding

No financial funding was received to conduct this study.
