# Supplementary material for: No correlation between thrombin generation and emicizumab levels: implications for monitoring emicizumab therapy
Source: Res Pract Thromb Haemost. 2024 Dec 17;9(1):102658. doi: 10.1016/j.rpth.2024.102658 (PMC11754509; doi:10.1016/j.rpth.2024.102658)
Supplement: Supplementary Material [file mmc1.pdf]

SUPPLEMENTARY FILES

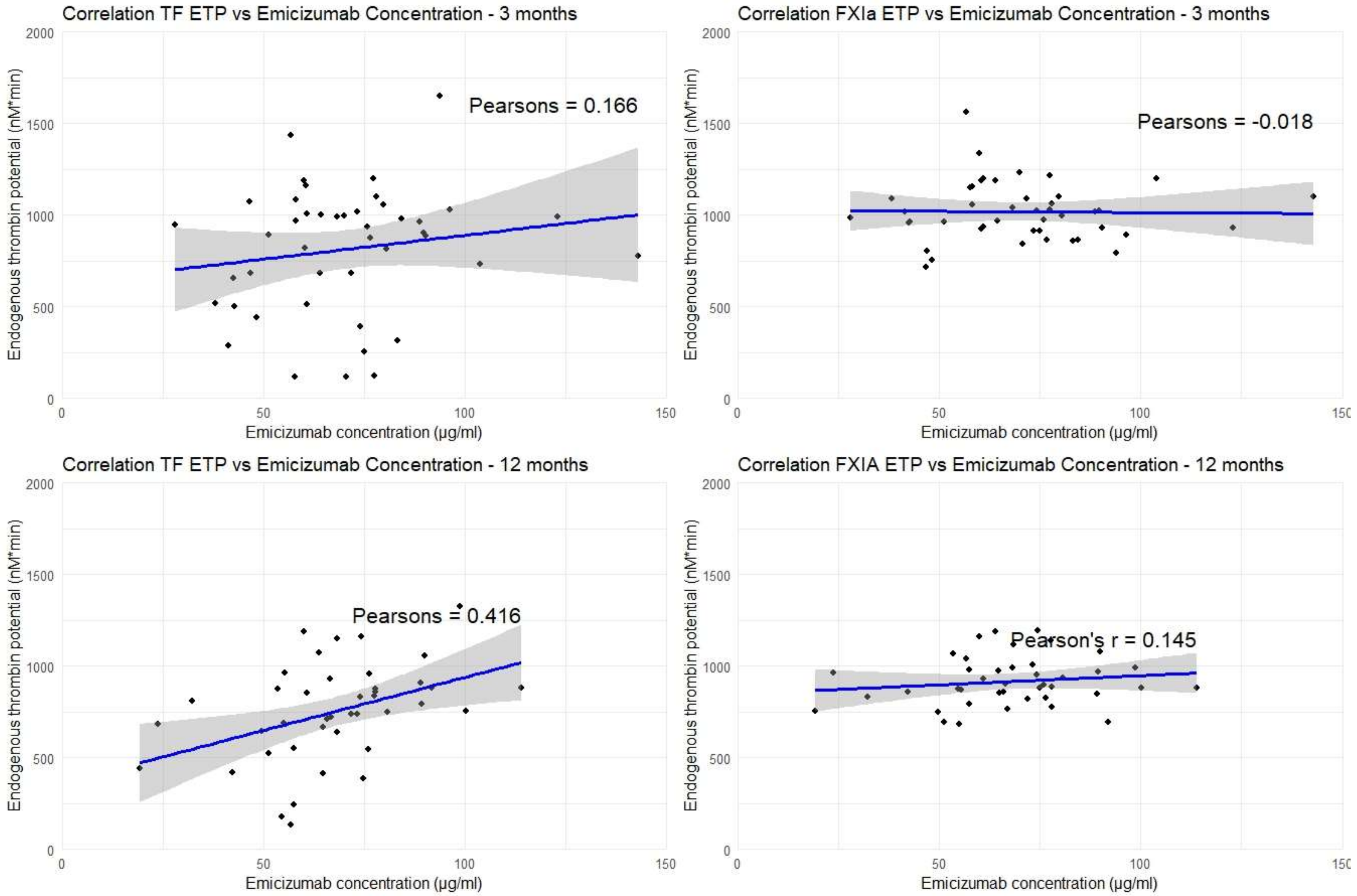

Figure S1: Correlation between emicizumab concentration and TF-ETP as well as FXIA-ETP at both three months and one year of therapy.

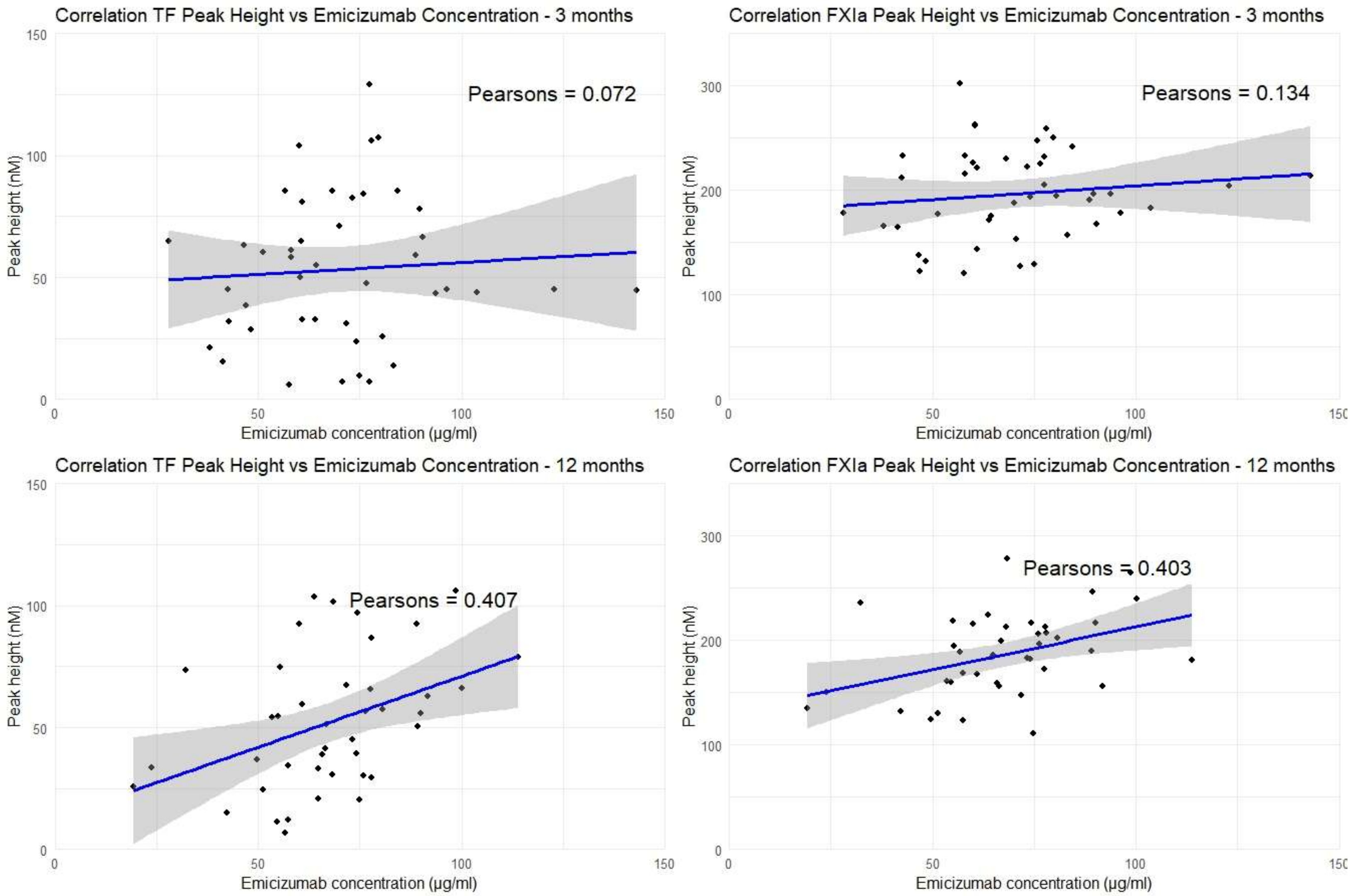

Figure S2: Correlation between emicizumab concentration and TF-Peak as well as FXIa-Peak at both three months and one year of therapy,

Table S1 Median and Interquartile ranges for all thrombin generation parameters triggered with Tissue Factor and Factor XIa at 3 months and 12 months of emicizumab therapy for Children and Adults.

**TG parameters**			
**T = 3 Months**	**Children**	**Adults**	**P-value[a]**
TF_ETP	804 (IQR 485 – 960)	966 (IQR 682 -1036)	0.178
TF_Peak	46 (IQR 31 – 64)	54 (IQR 30 – 83)	0.345
FXIa_ETP	961 (IQR 903 – 976)	1035 (IQR 925 – 1164)	0.044*
FXIA_Peak	178 (IQR 137 – 227)	201 (IQR 171 – 231)	0.197
**T = 12 Months**	**Children**	**Adults**	**P-value[a]**
TF_ETP	605 (IQR 406 – 821)	840 (IQR 691 – 966)	0.006*
TF_Peak	30 (IQR 19 – 57)	56 (IQR 39 – 79)	0.004*
FXIa_ETP	868 (IQR 829 – 868)	951 (IQR 821 – 1068)	0.151
FXIA_Peak	187 (IQR 153 – 207)	183 (IQR 156 – 217)	0.615

[a] Wilcoxon rank sum test.

Table S2: Proportion of bleeding and annualized (joint) bleeding rate

	**Overall (n=49)**	**Adults (n=33)**	**Children (n=16)**
**Proportion without treated bleeds - no %**			
Zero treated bleeds	30 (61)	22 (67)	8 (50)
≥ 1 treated bleeds	19 (39)	11 (33)	8 (50)
**Proportion without treated joint bleeds - no %**			
Zero treated bleeds	36 (73)	26 (79)	*10 (62)*
≥ 1 treated bleeds	13 (27)	7 (21)	*6 (38)*
**Annualized Bleeding Rate**			
Mean	0.6	0.4	0.8
95CI	0.4 – 0.9	0.2 – 0.8	0.5 – 1.5
**Annualized Joint Bleeding Rate**			
Mean	0.3	0.2	0.4
95CI	0.2 – 0.5	0.1 - 0.5	0.2 – 0.8

Results are presented number (%) or mean (95CI). Only one spontaneous bleed was observed.
